# Protective Effects of *Anthocleista djalonensis* Extracts against Pentylenetetrazole-Induced Epileptic Seizures and Neuronal Cell Loss: Role of Antioxidant Defense System 

**DOI:** 10.1155/2021/5523705

**Published:** 2021-08-30

**Authors:** Germain Sotoing Taiwe, Arielle Larissa Ndieudieu Kouamou, Bernard Dabole, Armelle Rosalie Mbang Ambassa, Hart Mann Alain Youbi Mambou, Raymond Bess Bila, Thierry Bang Tchoya, Joseph Renaud Menanga, Paul Desire Djomeni Dzeufiet, Elisabeth Ngo Bum

**Affiliations:** ^1^Department of Zoology and Animal Physiology, Faculty of Science, University of Buea, Buea, Cameroon; ^2^Department of Animal Biology and Physiology, Faculty of Science, University of Yaounde I, Yaounde, Cameroon; ^3^Department of Chemistry, Faculty of Science, University of Maroua, Maroua, Cameroon; ^4^Department of Biological Sciences, Faculty of Science, University of Ngaoundere, Ngaoundere, Cameroon; ^5^Department of Biological Sciences, Faculty of Science, University of Maroua, Maroua, Cameroon

## Abstract

Oxidative stress and neurodegeneration are involved in the initiation of epileptogenesis and progression of epileptic seizures. This study was aimed at investigating the anticonvulsant, antioxidant, and neuroprotective properties of active fractions isolated from *Anthocleista djalonensis* root barks in pentylenetetrazole mouse models of epileptic seizures. Bioactive-guided fractionation of *Anthocleista djalonensis* (AFAD) extracts using acute pentylenetetrazole (90 mg/kg) induced generalised tonic-clonic seizures, which afforded a potent anticonvulsant fraction (FPool 5). Further fractionation of AFAD was performed by high-performance liquid chromatography, which yielded fifteen subfractions, which were chemically characterised. In addition, AFAD was tested against convulsions or spontaneous kindled seizures induced, respectively, by acute (50 mg/kg) or subchronic (30 mg/kg) injection of pentylenetetrazole. Finally, oxidative stress markers, brain GABA content, and neuronal cell loss were evaluated in AFAD-treated pentylenetetrazole-kindled mice. Administration of AFAD significantly protected mice against acute pentylenetetrazole (90 mg/kg)-induced convulsions. In acute pentylenetetrazole (50 mg/kg)-induced hippocampal and cortical paroxysmal discharges, AFAD significantly decreased the number of crisis, the cumulative duration of crisis, and the mean duration of crisis. Additionally, AFAD significantly decreased the number of myoclonic jerks and improved the seizure score in subchronic pentylenetetrazole-induced kindled seizures. The pentylenetetrazole-induced alteration of oxidant-antioxidant balance, GABA concentration, and neuronal cells in the brain were attenuated by AFAD treatment. This study showed that AFAD protected mice against pentylenetetrazole-induced epileptic seizures possibly through the enhancement of antioxidant defence and GABAergic signalling. These events might be correlated with the amelioration of neuronal cell loss; hence, AFAD could be a potential candidate for the treatment of epilepsy.

## 1. Introduction

Grand mal seizures are a complex type of neurological disorders commonly found in the tropical area of Africa and are characterised by an abnormal, hypersynchronous discharger of a population of neurons in the brain that can lead to a loss of consciousness, violent muscle contractions, and eventually oxidative stress leading to the neuronal cell loss [1, 2]. There are a significant number of studies indicating strong evidence linking kindled seizures, epileptogenesis, and oxidative stress leading to a neuronal cell loss [3, 4]. A reduction of antioxidant defence in the central nervous system directly affects glutamate receptor activation and GABAergic signalling in neurons, leading to neurotransmission dysfunction in the brain, described by the term excitotoxicity [5]. More so, excitotoxicity, accumulation of lipid peroxidation, and the increase of DNA and protein damages encourage oxidative stress and are associated with an imbalance of neuronal excitation and inhibition and the activation of excitable neural network. The disturbance occurring in the epileptic seizures is caused by electrical signals spreading through the brain inappropriately [6, 7]. The principal contemporary therapeutic approach to epilepsy is the chronic administration of anticonvulsant drugs to inhibit the occurrence of seizures. This therapy is often unsatisfactory since seizures persist in at least 30% of patients and undesirable side effects are common [8]. In patients who have a brain injury associated with a high risk of epilepsy occurring months to years later, it may be possible to intervene pharmacologically to prevent epileptogenesis and eventually epilepsy [9–12]. No antiepileptic drug is free from adverse effects, but there are significant differences in type, frequency, and duration of adverse effects caused by individual medications [13]. Today, with about 26 different medications available, selection of an antiepileptic drug is a much more complex process [14]. These drugs have better safety records, but all are liable to produce adverse effects that interfere with quality of life [15]. Natural products have become an important source of great interest and value in the search of new drugs and lead compounds for the treatment of epilepsy [16].

*Anthocleista djalonensis* A. Chev. (Loganiaceae) is a medicinal plant widely distributed in the northern Cameroon, traditionally used for the treatment of epilepsy and associated morbidities, such a depression, anxiety, and insomnia [17–20]. According to Cameroonian traditional healers, root barks are the preferred part of *Anthocleista djalonensis* used for the treatment of epilepsy, infantile convulsions, memory impairment, anxiety, depression, and agitation [21–23]. Previous studies performed on qualitative phytochemical analysis of this plant indicated the presence of alkaloids, flavonoids, triterpenes, and phenolic compounds in the root barks and leaves [19, 24–27] in which several classes of compounds are identified (phthalide, xanthone, monoterpene diol, iridoid glucoside, and pentacyclic triterpene). In the past decades, many of the families and classes of compounds have received a great attention owing to their biological properties, including anticonvulsant, antioxidative, immunoregulative, and anti-inflammatory properties [27, 28]. In order to understand the mechanisms of action of *Anthocleista djalonensis* during epileptogenesis and/or epileptic seizures, we isolated some active fractions from the root barks of *Anthocleista djalonensis* using solvent-guided fractionation and bioassay-guided studies. Hence, this study was carried out to investigate the protective effect of active fractions isolated from *Anthocleista djalonensis* on pentylenetetrazole models of generalised tonic-clonic convulsions, oxidative stress, GABAergic deficits, and neuronal cell loss in the brain of mice.

## 2. Materials and Methods

### 2.1. Plant Collection and Authentication

The root barks of *Anthocleista djalonensis* were harvested in March 2016 from the Mount Tenglin area of Pitoa, North Region of Cameroon (with the harvesting coordinates 9°63′12″ N and 13°21′4″ E). The specimen is preserved with a voucher specimen number 41786/HNC at the National Herbarium of Yaounde, Cameroon.

### 2.2. Solvent-Guided Fractionation of *Anthocleista djalonensis* Extracts

The barks of the dried root of *Anthocleista djalonensis* were ground in a mill, and 1000 g of powder was obtained and put for maceration in 5000 mL of distilled water. After 7 hours, the macerate was boiled for 20 min. The supernatant was collected after cooling and filtered using Whatman number 1 filter paper. The extract was evaporated *in vacuo*, and 102.86 g aqueous extract was obtained. The yield of the extraction was 10.28%. After dissolution of residue in 500 mL warm water for one hour, the mixture was successively extracted with ethyl acetate (0.5 *l* × 3) and n-butanol (0.5 *l* × 3) to give two residues. The second residue 175.92 mg was resolved again in 1000 mL warm water, acidified with 1 N HCl (pH 4–5), and extracted three time with 500 mL CHCl_3_. Finally, NaOH (1 N), pH 9–10, was used to neutralise the aqueous phase and the mixture was extracted three times again with CHCl_3_. After concentration *in vacuo*, 98.43 g of crude and brown extracts were collected.

### 2.3. Bioassay-Guided Studies of *Anthocleista djalonensis* Extracts

The crude extract of *Anthocleista djalonensis* was chromatographed using high-performance liquid chromatography (HPLC, Shimadzu-LC 20AT). For elution of the plant extract, a gradient of two solvents was used, A and B. The mobile phase consisted of solvent A (water) and solvent B (acetonitrile). This method of separation was performed on TC-C18. The elution was undertaken using a linear gradient of 25–100% of B in 60 min. The temperature of column was maintained at 30°C with a stable flow rate (1.0 mL/min), and solutions were injected at a volume of 20 *μ*L. The sample was dissolved in a mixture of acetonitrile and water (30 : 70, v/v). Detection was performed at 280 nm, and veratraldehyde was used as an internal standard. All the compounds were identified by comparison with the authentic chromatographic standards available [24–26] in our laboratory and according to the method previously reported [8]. After elution, 148 fractions were collected, pooled, and lyophilised to give FPool 1 (25.17 mg), FPool 2 (31.27 mg), FPool 3 (21.69), FPool 4 (19.84 mg), FPool 5 (36.92 mg), FPool 6 (28.75 mg), FPool 7 (30.46 mg), FPool 8 (27.14 mg), FPool 9 (26.42 mg), and FPool 10 (18.57 mg). Thereafter, the pooled fractions were submitted to the bio-guided assay using 90 mg/kg pentylenetetrazole-induced tonic-clonic convulsions in mice. Interestingly, FPool 5 (containing FI (2.29 mg), FII (3.19 mg), FIII (2.42 mg), FIV (1.91 mg), FV (2.29 g), FVI (2.02 mg), FVII (2.21 mg), FVIII (3.41 mg), FIX (2.37 mg), FX (2.87 mg), FXI (2.49 mg), FXII (2.13 mg), FXIII (3.38 mg), FXIV (1.82 mg), and FXV (2.12 mg)) significantly antagonised tonic-clonic convulsions induced by 90 mg/kg pentylenetetrazole and strongly protected mice against death compared with the negative control group.

**Figure 1:**
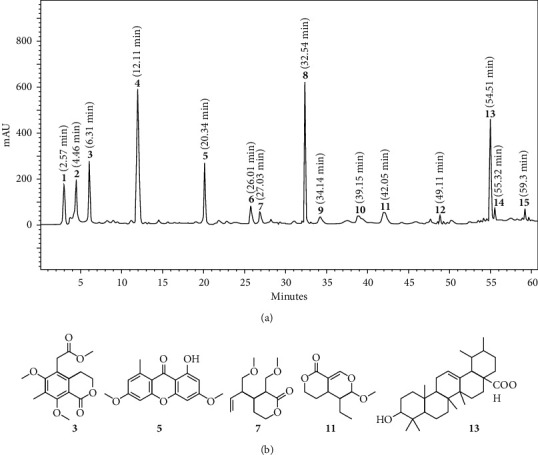
Some active constituents of *Anthocleista djalonensis*: (a) HPLC profile of the standardised active fractions from *Anthocleista djalonensis*. (b) Chemical structures of major compounds identified from the standardised active fractions: 1, djalonensin; 2, lichexanthone; **3**, djalonenol; 4, djalonenoside; a, internal standard (veratraldehyde); and **5**, ursolic acid. AFAD is constituted with the compounds **3**, **5**, **7**, **11**, and **13**, respectively, from the classes of phthalide, xanthone, monoterpene diol, iridoid glucoside (sweroside), and pentacyclic triterpene.

Phytochemical analysis using standard compounds demonstrated that fractions FIII, FV, FVII, FXI, and FXIII contained in the pooled fractions (FPool 5) were described in previous studies [24, 25]. The purity and structures of isolated compounds were confirmed using standards (97%) by HPLC, ^13^C NMR, and ^1^H-NMR [24]. The purity of each fraction FIII, FV, FVII, FXI, and FXIII was 78.3%, in which djalonensin (FIII or (3)) accounted for 16%, lichexanthone (FV or (5)) 18%, djalonenol (FVII or (7)) 11%, djalonenoside (FXI or (11)) 31%, and ursolic acid (FXIII or (13)) 24% (Figure 1), and the yield of extraction was 0.00127%. The standardised pooled active fractions of *Anthocleista djalonensis* (AFAD) was dissolved in 0.9% saline (containing 2% dimethyl sulfoxide), administered orally to mice with a volume of 10 mL/kg.

### 2.4. Chemicals

Chloral hydrate, cresyl violet, clonazepam, ethyl acetate, ethylenediaminetetraacetic acid, glutamic acid, n-butanol, nicotinamide adenine dinucleotide, nitro blue tetrazolium, N-naphthyl ethylene diamine, pentylenetetrazole (PTZ), pyridine, pyridoxal phosphate, sodium dodecyl sulphate, sodium chloride, sulfanilamide, thiobarbituric acid, tris-HCl, *α*-oxoglutarate, and 5′5-dithiobis (2-nitrobenzoic acid) were purchased from Sigma Chemical Corporation, St. Louis, USA. Sodium valproate was obtained from SANOFI-AVENTIS, Gentilly, France. Ketamine is provided by ROTEXMEDICA GmbH, Trittau, Germany. Xylazine was obtained from Prodivet pharmaceuticals sa/nv, Raeren, Belgium. All the others used chemicals were from Sigma Chemical Corporation, St. Louis, USA.

### 2.5. Animals and Ethical Aspects

Adult Swiss mice weighting 20–25 g were obtained from the National Veterinary Laboratory, Garoua, Cameroon, and used throughout these studies. They were housed in standard Plexiglas cages with food and water *ad libitum*. The animal house was maintained constantly at 25°C on a 12-h light-dark cycle. The protocols were performed in concordance with the International Guide for the Care and Use of Laboratory Animal (National Institute of Health; publication No. 85-23, revised 1996) and the Cameroon National Ethical Committee, Yaounde (No. FW-IRB00001954) and also received approval from the Institutional Ethical Committee (UB-IACUC No 002/2019).

### 2.6. Acute Administration of Pentylenetetrazole-Induced Tonic-Clonic Convulsions

Tonic-clonic convulsions were induced in mice by intraperitoneal injection of 90 mg/kg pentylenetetrazole [29]. One group of ten mice received vehicle (negative control group) at a volume of 10 mL/kg orally, and another group received intraperitoneally 0.1 mg/kg clonazepam (positive control group). The other four groups of ten mice each received the four different doses of AFAD (20, 40, 80, and 160 mg/kg) orally. One hour later, any behavioural changes related to convulsion or death were carefully observed for 30-min duration in all the animals.

### 2.7. Acute Administration of Pentylenetetrazole-Induced Seizures and *Exitus*

#### 2.7.1. Surgery

Six groups of six mice were used for acute 50 mg/kg pentylenetetrazole-induced seizures and death (fatal termination of seizures). They were anesthetised using intraperitoneal injection of ketamine (75 mg/kg) and xylazine (8 mg/kg). Immediately, they were placed into a stereotaxic frame for surgery and implanted stereotaxically with bipolar electrodes inserted into the hippocampus (anteroposterior: −1.8, mediolateral: −1.9, dorsoventral: −1.8 mm) with the bregma as a reference [8]. Two other monopolar electrodes were placed over the right and left frontoparietal cortex (AP = +1.4 mm, ML = −1.6 mm, DV = −2 mm from the bregma). One reference electrode (a monopolar electrode) was implanted over the cerebellum [30]. Finally, the implanted electrodes were assembled to the mice skull using dental acrylic. At the end of experiment, verification of electrodes implantation into the brain was performed using histology and tissues were stained with cresyl violet as previously described [8, 31].

#### 2.7.2. Electroencephalographic Recording and Treatment of Mice

Electroencephalograms were recorded using a Biopac MP160 system—Biopac Systems Inc., Goleta, CA—equipped with a Faraday cage. The recorded signals were amplified, filtered, and digitised (256 Hz) using AcqKnowledge Software 5.0. Each mouse was individually recorded in a compartment. The entire EEG recording did not exceed 3 hours, given 45–60 min for habituation of the study setup. Excitation, myoclonus, and clonic seizures were recorded continuously in the study animals. One group of six mice received vehicle at a volume of 10 mL/kg orally and served as a negative control group; another group of six animals received 1 mg/kg clonazepam. Test groups of mice were treated with AFAD at the doses of 20, 40, 80, and 160 mg/kg. Animals were mounted on the EEG acquisition system for a period of 60 min recording. After the initial recording, seizures were induced by intraperitoneal injection of 50 mg/kg pentylenetetrazole and the mice were recorded again for 120-min duration. The number of crisis, the mean duration of crisis, and the cumulative duration of crisis were observed and noted [8, 31].

### 2.8. Chronic Administration of Pentylenetetrazole-Induced Kindled Seizures

#### 2.8.1. Experimental Design

Pentylenetetrazole (30 mg/kg; i.p.) and AFAD (20, 40, 80 and 160 mg/kg; *per os* (*p.o.*)) were coadministered in different groups of mice. The treatments were repeated until seizure stage 5. Two groups of animals were added and treated with 10 mL/kg vehicle or 300 mg/kg sodium valproate. Pentylenetetrazole was given 1 hour after the administration of the standardised fractions. The other group of mice was coadministered with vehicle (*p.o.*) and 0.9% saline (i.p.) and served as a normal group. The last group of animals was treated with 160 mg/kg AFAD to study the effects of the standardised fraction administered alone. Animals were observed for their behaviour one day following the last pentylenetetrazole administration. The duration time was 30 min for each animal, and seizure severity was scored from 0 to 5 [8, 32, 33].

#### 2.8.2. Kindling Induction and Treatment

Spontaneous kindled seizures were induced in normal mice by 22 injections on every two alternated days. This procedure was described previously by Taiwe et al. [33]. Seizure severity for each mouse was evaluated using Racine's scale [34]. Finally, the latency (or onset time to the seizures) and number of generalised tonic-clonic seizures were quantified [8, 33]. At the end of behavioural evaluations, all the animals were euthanised by inhalation of high concentration of compressed carbon dioxide (CO_2_) gas in cylinders, and the whole brain was collected for biochemical and histological analyses [4].

#### 2.8.3. Biochemical Analysis after Chronic Administration of Pentylenetetrazole-Induced Kindled Seizures

*(1) Evaluation of Brain GABA Concentration and Determination of GABA Transaminase Activity*. Concentration of GABA in the brain homogenates was quantified as described previously by Lowe et al. with slight modifications [35]. This concentration was expressed in µg/g of wet brain tissue [36].

*(2) Determination of GABA Transaminase Activity*. The brains were removed and submerged in ice-cold artificial cerebrospinal fluid. Briefly, the brain tissue of each mouse was then washed to remove blood, blotted to dry and submerged in 5 mL of methanol, homogenised using a glass Teflon homogeniser for 2 min, and centrifuged at 10,000 rpm at −10°C for 15 min [37]. Finally, GABA-T activity was quantified in the brain homogenates spectrophotometrically as described previously [37, 38] and modified by Taiwe et al. [32].

*(3) Lipid Peroxidation Assay*. Malondialdehyde (MDA), an indicator of lipid peroxidation, was determined in the brain homogenates (20%). Briefly, the brain homogenate supernatant (500 *µ*L) was added to 250 *µ*L of trichloroacetic acid solution (20%) and 500 *µ*L of thiobarbituric acid solution (0.67%). The mixture was incubated at 90°C for one hour and then cooled with tap water, and malondialdehyde was estimated spectrophotometrically as described by Liu et al. [39].

*(4) Measurements of Glutathione Levels*. The reduced glutathione (GSH) was estimated spectrophotometrically by the method described by Ellman (Ellman, 1959). The brain homogenates were prepared following this previous protocol, and finally, the absorbance of the mixture was determined at 412 nm. The concentration of reduced glutathione was expressed as µg/g tissue [8].

*(5) Superoxide Dismutase Activity Determination*. Total superoxide dismutase (SOD) activity was evaluated according to the method of Sun et al. [40]. After preparation and mixture of each brain homogenate as described in this protocol, the SOD activity was estimated as the enzyme amount causing 50% inhibition in the nitroblue tetrazolium reduction rate [4].

*(6) Measurements of Nitric Oxide*. The total nitrite was estimated spectrophotometrically by the method described by Cortas and Wakid [41]. The principle of this method is based on the conversion of nitrate into nitrite by cadmium and followed by colour development with Griess reagent (1% sulfanilamide and 0.1% N-naphthyl ethylene diamine) in an acidic medium (2.5%). The reference was a sodium nitrate dose-response curve. The absorbance of each brain homogenate was quantified at 570 nm [4].

#### 2.8.4. Histological Analysis

In order to determine viable and nonviable neuronal cells in pentylenetetrazole-challenged mice, Nissl staining was performed at the end of the behavioural evaluations [42]. Fully kindled animals were sacrificed by inhalation of high concentration of compressed carbon dioxide (CO_2_) gas in cylinders, and the whole brain was collected for histological analyses. Thereafter, each animal was transcardially perfused with 100 mL NaCl (0.9%). Animals were additionally perfused with 50 mL paraformaldehyde prepared in 0.05 M sodium phosphate (pH 7.4, containing 0.8% NaCl). Finally, the brains were isolated and fixed in 4% paraformaldehyde at 4°C for 24 hours. Rehydration of each tissue was performed by upgraded series of 40, 60, 70, 80, 90, and 100% ethanol for 60-min duration each. Thereafter, the samples were cleared using xylene and finally embedded in paraffin. Serial coronal sections with 10 *μ*m thickness include hippocampal CA1, CA3, and dentate gyrus subregions and were performed using a microtome. In addition, paraffin was removed from the coronal section using xylene, and using series of downgraded hydration (100, 95, 70, and 50% ethanol; 5 min), each coronal section was hydrated. The sections were rinsed for 5 min in distilled water, mounted on gelatin-coated slides for staining. Thereafter, preparations were immersed in 0.5% cresyl violet prepared in 90 mM acetic acid and 10 mM sodium acetate for 10–30-min duration, until the desired depth of staining was achieved. Additionally, the sections were rinsed in distilled water, dehydrated in solutions of ascending concentration of 75, 90, and 100% ethanol, cleared in xylene, and then protected with a coverslip. Captures and quantitative analysis of cell numbers of the CA1, CA3, and DG hilus subregions of the hippocampus from each mouse were performed using Image *J* software (NIH, USA). For each photographed coronal section, six visual fields of 0.6 mm^2^ were realised, and the numbers of stained neuronal cells per high-power field were counted at a higher magnification (×400).

### 2.9. Acute Toxicity Test

To determine the median effective dose (ED_50_) of AFAD, we administered acutely and gradually different doses of AFAD in male and female mice, separately. Immediately after the oral administration of AFAD (20, 40, 80, 160, 320, 640, 1280, 2560, 5120, and 7680 mg/kg, orally) or vehicle at a volume of 10 mL/kg, each mouse was allowed access to water and food *ad libitum* and the incidence of convulsion, sedation, rearing, grooming, increased or decreased of respiration, hyperactivity, loss of righting reflex, food and water intake, and mortality were observed. All the animals were observed also for a period of 14 days [43, 44]. The median lethal dose (LD_50_) was determined by the method described by Litchfield and Wilcoxon [45].

### 2.10. Statistical Analysis

Data are shown as mean ± standard error of the mean (S.E.M.) or as percentages of protection of mice. The dose necessary to reduce the response of mice by 50% relative to the negative control value (ED_50_) and 95% confidence intervals values were determined. Statistical differences between control and treated groups were tested by one-way ANOVA followed by Bonferroni's multiple comparison test. *P* values less than 0.05 were considered significant.

## 3. Results

### 3.1. Chemical Analysis of the Standardised Active Fractions from *Anthocleista djalonensis*

The barks of dried root of *Anthocleista djalonensis* were initially extracted using organic solvents to afford 98.43 g of crude extract. Furthermore, the crude extract was chromatographed with high-performance liquid chromatography, and 148 fractions were obtained. The fractions were pooled and tested for their anticonvulsant activities using 90 mg/kg pentylenetetrazole-induced tonic-clonic convulsions. The more potent anticonvulsant fractions (FPool 5) were selected based on the highest latency to convulsion (20.84 ± 01.82), highest % surviving animals (20%), and lowest percentage of mice exhibiting convulsion (70%) (Table 1) and pooled to afford standardised active fractions from *Anthocleista djalonensis* (AFAD) constituted with fifteen chemical compounds in which some of them were identified. The purity and structures of isolated compounds were confirmed using standards (97%) by HPLC, ^13^C NMR, and ^1^H-NMR. Chemical structures of major compounds identified from AFAD were as follows: 1, djalonensin; 2, lichexanthone; 3, djalonenol; 4, djalonenoside; a, internal standard (veratraldehyde); and 5, ursolic acid. AFAD is constituted with the compounds 3, 5, 7, 11, and 13, respectively, from the classes of phthalide, xanthone, monoterpene diol, iridoid glucoside (sweroside), and pentacyclic triterpene. The purity of each fraction FIII, FV, FVII, FXI, and FXIII was 78.3% (Figure 1).

### 3.2. Effects of AFAD on Tonic-Clonic Convulsions Induced by Acute Administration of Pentylenetetrazole

Treatment of mice with the pooled fractions FPool 1, FPool 2, FPool 3, FPool 4, FPool 6, FPool 7, FPool 8, FPool 9, and FPool 10 at a dose of 80 mg/kg did not affect the occurrence of convulsions induced by pentylenetetrazole. However, the fraction FPool 1 administered orally to mice at a dose of 80 mg/kg significantly protected animals against convulsions induced by pentylenetetrazole. In control experiments, oral administration of pentylenetetrazole 90 mg/kg produced systematically tonic-clonic convulsions in all the animals (100%). Active pooled fraction from *Anthocleista djalonensis* significantly decreased the percentage of mice exhibiting convulsions, increased the latency to convulsion and death, and increased the percentage of surviving animals. AFAD was administered orally at the appropriate doses (20, 40, 80, and 160 mg/kg) as indicated in various experiments. Administration of AFAD at the doses of 40, 80, and 160 mg/kg significantly protected [*F* (15, 144) = 471,45; *P* < 0.001] mice against tonic-clonic convulsions induced by pentylenetetrazole. AFAD increased the latency to convulsions from 03.27 ± 01.13 min to 21.64 ± 03.81 min (*P* < 0.001) at a dose of 80 mg/kg and from 03.27 ± 01.13 min to 26.71 ± 01.19 min at a dose of 160 mg/kg. Administration of AFAD at the doses of 80 and 160 mg/kg protected 80% (*P* < 0.001) and 90% (*P* < 0.001) of mice against convulsions induced by 90 mg/kg pentylenetetrazole, respectively. In addition, 90% of mice that were administered AFAD at 160 mg/kg survived compared with the vehicle-treated pentylenetetrazole mice (no surviving animal). AFAD showed potent and dose-dependent protection of generalised tonic-clonic seizures with an ED_50_ value (95% CI) of 37.51 (31.14–42.28) mg/kg (Table 1).

### 3.3. Effects of AFAD on Seizures and *Exitus* Induced by Acute Administration of Pentylenetetrazole

**Figure 2:**
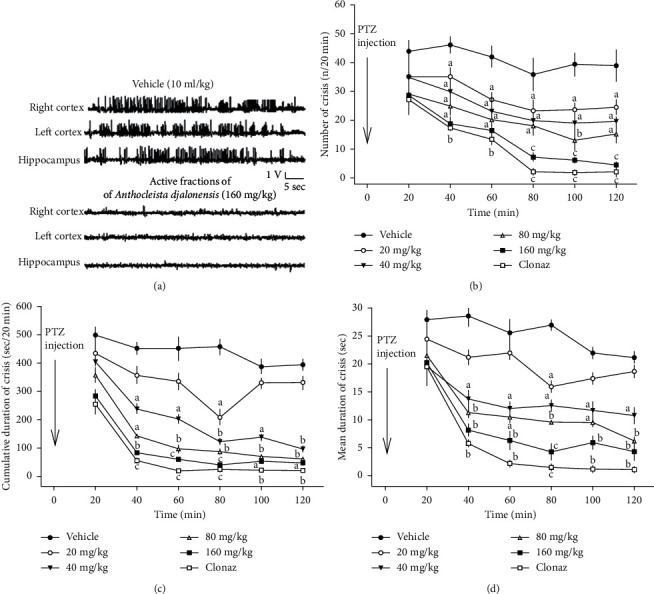
Effects of AFAD on acute administration of pentylenetetrazole-induced seizures in mice: *panel a*: hippocampal and cortical paroxysmal discharges, *panel b*: number of crisis (n/20 min), *panel c*: cumulative duration of crisis (sec/20 min), and *panel d*: mean duration of crisis (sec). *N* = 6 animals per dose. Data were analysed by two-way repeated measures analysis of variance followed by the Newman–Keuls post hoc test; ^a^*P* < 0.05, ^b^*P* < 0.01, ^c^*P* < 0.001, significantly different compared with the vehicle; Clonaz, clonazepam 0.1 mg/kg.

Characteristics of behavioural seizures with paroxysmal EEG were observed rapidly in 50 mg/kg pentylenetetrazole-challenged mice. In the negative control group constituted with the mice treated with vehicle, the latencies to the first myoclonic seizures (whole body jerk) and *exitus* (motor seizures) were close to the first minute. The animals exposed to pentylenetetrazole showed one or two tonic-clonic seizures, which lasted around two and three minutes. The clonic seizure is characterised by the rapid alternation of muscle contraction and/or relaxation immediately followed by a tonic seizure. The tonic seizure (hind limb extension) was observed between two or three episodes of clonic seizures in some animals treated with vehicle and challenged with 50 mg/kg pentylenetetrazole. In addition, these behavioural seizures associated with head nodding were observed concomitantly with hippocampal and cortical paroxysmal spike-and-wave discharges lasted between fifteen and sixty seconds (Figure 2(a)). Their rate of occurrence was variable between the negative control group and tested groups, with a maximum of one discharge every other minute. In some cases, high-voltage spike-and-wave (1500–4500 *µ*V, 3–5 Hz) discharges followed by low-voltage rhythmic activity and higher frequency (10–14 Hz, 700–1100 *µ*V) were recorded 20 min in the vehicle-treated pentylenetetrazole group (Figure 2(a)). EEG activity was strongly impaired, and mice showed signs of motor incapacitation or alteration of motor coordination. A high-voltage fast epileptiform activity associated with isolated spike-and-wave discharges is recorded in all the vehicle-treated pentylenetetrazole mice. Oral administration of AFAD in a dose-dependent manner protected animals against *exitus*, generalised tonic-clonic seizures. AFAD completely inhibited the EEG power spectrum changes evoked after the pentylenetetrazole administration. The doses of 80 and 160 mg/kg AFAD significantly inhibited hippocampal and cortical paroxysmal discharges in a dose-dependent manner. AFAD strongly antagonised the number of crisis [*F* (5, 30) = 517.42; *P* < 0.001], the cumulative duration of crisis [*F* (5, 30) = 85.34; *P* < 0.001], and the mean duration of crisis [*F* (5, 30) = 209.42; *P* < 0.001] compared with the vehicle-treated pentylenetetrazole (Figures 2(b)–2(d)). These effects of 80 and 160 mg/kg AFAD were comparable with those of a standard antiepileptic drug, clonazepam administered at 0.1 mg/kg. AFAD efficiently inhibited with an ED_50_ value (95% CI) of 36.51 (30.75–38.12) mg/kg (Figure 2(a)).

### 3.4. Effects of AFAD on Induction of Kindling by Chronic Administration of Pentylenetetrazole

#### 3.4.1. Effects of AFAD on Kindled Seizures

**Figure 3:**
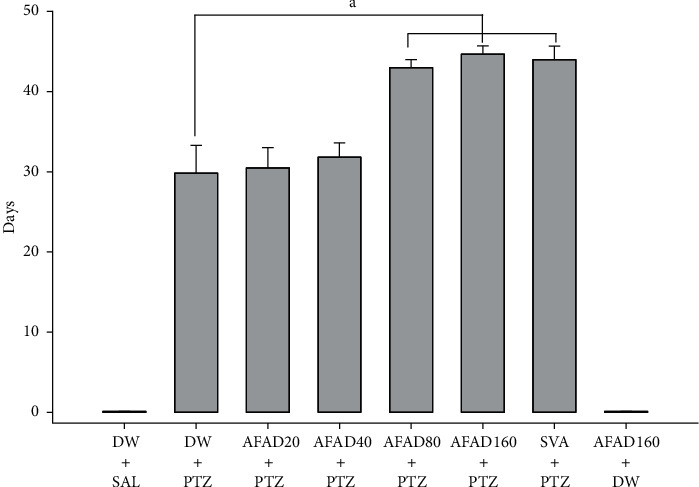
Development of pentylenetetrazole-evoked kindling in mice pretreated 1 h before each kindling session with vehicle, AFAD, or sodium valproate. Results are expressed as mean ± S.E.M. for 6 animals. Data were analysed by one-way ANOVA followed by Bonferroni's multiple comparison test; ^a^*P* < 0.05 significantly different compared with the pentylenetetrazole-challenged mice. AFAD20, standardised active fractions from *Anthocleista djalonensis* 20 mg/kg; DW, distilled water; PTZ, 30 mg/kg pentylenetetrazole; SVA, 300 mg/kg sodium valproate; SAL, saline.

The results obtained in vehicle-treated pentylenetetrazole indicated the development of kindling in 29.83 ± 3.44 days. The standardised extract of *Anthocleista djalonensis* (80 and 160 mg/kg, p.o.) treatment dose dependently decreased the incidence of kindling development in animals [*F* (7, 40) = 704.19, *P* < 0.05]. The treatment with a standard antiepileptic drug, sodium valproate (300 mg/kg), in kindled mice also showed a significant reduction in the incidence and severity of seizures. The results of protection offered by AFAD 160 mg/kg were comparable with those of the sodium valproate-treated group (Figure 3).

**Figure 4:**
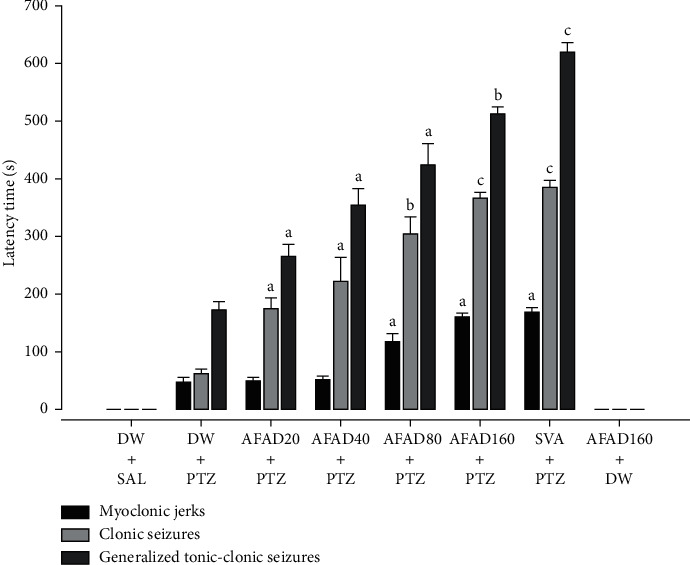
Effects of AFAD or sodium valproate on the latencies to myoclonic jerks, clonic seizures, and generalised tonic-clonic seizures in pentylenetetrazole-kindled mice. Results are expressed as mean ± S.E.M. for 6 animals. Data were analysed by one-way ANOVA followed by Bonferroni's multiple comparison test; ^a^*P* < 0.05, ^b^*P* < 0.01, and ^c^*P* < 0.001, significantly different compared with the pentylenetetrazole-challenged mice. AFAD20, standardised active fractions from *Anthocleista djalonensis* 20 mg/kg; DW, distilled water; PTZ, 30 mg/kg pentylenetetrazole; SVA, 300 mg/kg sodium valproate; SAL, saline.

The results showed that the oral administration of AFAD induced a significant and dose-dependent increase in the latency of myoclonic jerks [*F* (7, 40) = 431.75, *P* < 0.001] as well as the latency to clonic seizures [*F* (7, 40) = 109.42, *P* < 0.001] and generalised tonic-clonic seizure [*F* (7, 40) = 95.61, *P* < 0.001] (Figure 4). AFAD produced a significant increase in the latency to myoclonic jerks from 46.01 ± 10.01 seconds in the vehicle-treated pentylenetetrazole mice to 116.83 ± 14.11 seconds (*P* < 0.05) and 161.81 ± 5.67 seconds (*P* < 0.05) in the test groups administered 80 and 160 mg/kg doses of AFAD, respectively. These results showed an obvious anticonvulsive effect for AFAD (Figure 4). Oral administration of AFAD also induced a significant increase in the latency to clonic seizures. This time increased from 61.83 ± 8.72 seconds in the vehicle-treated pentylenetetrazole mice to 222.50 ± 41.08 seconds (*P* < 0.05), 304.01 ± 29.67 seconds (*P* < 0.01), and 365.33 ± 11.59 seconds (*P* < 0.001) in pretreated animals with 40, 80, and 160 mg/kg doses of AFAD, respectively. The results indicated that the latency to clonic seizures was significantly increased from 61.83 ± 8.72 seconds in the vehicle-treated pentylenetetrazole mice to 222.50 ± 41.08 seconds (*P* < 0.05), 304.01 ± 29.67 seconds (*P* < 0.01), and 365.33 ± 11.59 seconds (*P* < 0.001) in pretreated mice with 40, 80, and 160 mg/kg doses of AFAD, respectively (Figure 4). There is a significant variation of the latency of generalised tonic-clonic seizures in the pretreated group of mice with AFAD. The latency to the generalised tonic-clonic seizures significantly increased from 172.50 ± 13.33 seconds in vehicle-treated pentylenetetrazole mice to 354.01 ± 28.33 seconds (*P* < 0.05), 423.17 ± 32.22 seconds (*P* < 0.05), and 513.47 ± 11.78 seconds (*P* < 0.01) in the groups administered 40, 80, and 160 mg/kg doses of AFAD, respectively (Figure 4).

**Figure 5:**
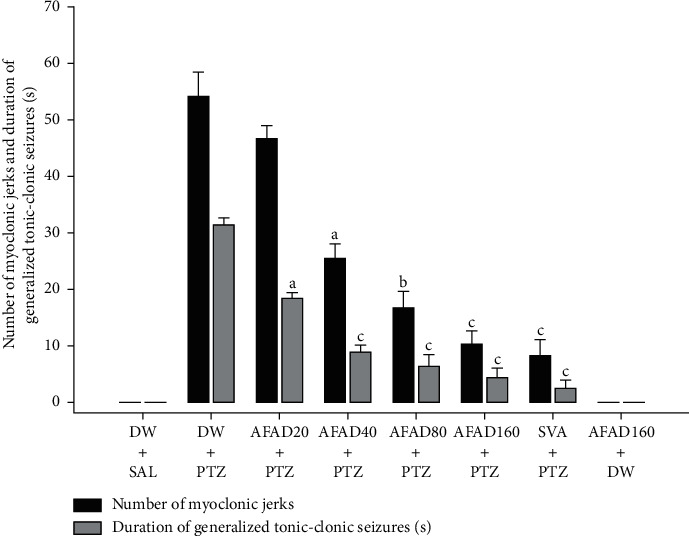
Effects of AFAD or sodium valproate on the number of myoclonic jerks and the duration of tonic-clonic seizures in pentylenetetrazole-kindled mice. Data were analysed by one-way ANOVA followed by Bonferroni's multiple comparison test; ^a^*P* < 0.05, ^b^*P* < 0.01, and ^c^*P* < 0.001, significantly different compared with the pentylenetetrazole-challenged mice. AFAD20, standardised active fractions from *Anthocleista djalonensis* 20 mg/kg; DW, distilled water; PTZ, 30 mg/kg pentylenetetrazole; SVA, 300 mg/kg sodium valproate; SAL, saline.

The number of myoclonic jerks are significantly different [*F* (7, 40) = 217.29, *P* < 0.001] between the vehicle-treated pentylenetetrazole mice and the mice administered different doses of AFAD. This number is decreasing from 54.15 ± 4.38 in the vehicle-treated pentylenetetrazole mice to 25.48 ± 2.53 (*P* < 0.05), 16.72 ± 2.74 (*P* < 0.01), and 10.25 ± 2.28 (*P* < 0.001) in the groups of mice administered AFAD 40, 80, and 160 mg/kg, respectively. In addition, the oral administration of AFAD significantly decreased the duration of generalised tonic-clonic seizures from 31.47 ± 1.18 seconds in the vehicle-treated pentylenetetrazole mice to 8.83 ± 1.29 seconds (*P* < 0.05), 6.37 ± 1.94 seconds (*P* < 0.01), and 4.17 ± 1.38 seconds (*P* < 0.01) for the doses 40, 80, and 160 mg/kg of AFAD, respectively (Figure 5).

**Figure 6:**
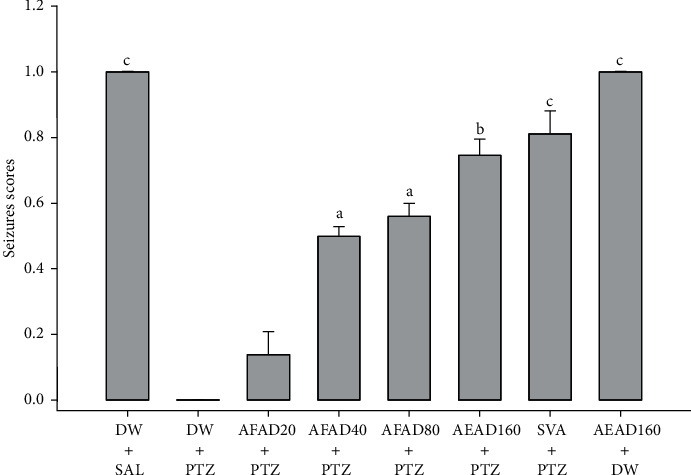
Effects of AFAD or sodium valproate on the seizure scores in pentylenetetrazole-kindled mice. Results are expressed as mean ± S.E.M. Data were analysed by one-way ANOVA followed by Bonferroni's multiple comparison test, ^a^*P* < 0.05, ^b^*P* < 0.01, and ^c^*P* < 0.001, significantly different compared with the pentylenetetrazole-challenged mice. AFAD20, standardised active fractions from *Anthocleista djalonensis* 20 mg/kg; DW, distilled water; PTZ, 30 mg/kg pentylenetetrazole; SVA, 300 mg/kg sodium valproate; SAL, saline.

Pretreatment of mice with vehicle was not able to avoid pentylenetetrazole-induced convulsions, and thus no significant increase in the seizure scores was observed in the vehicle-treated pentylenetetrazole mice. Interestingly, significant increases in the seizure scores were observed in the groups of mice administered AFAD 40, 80, and 160 mg/kg, respectively, as related to the vehicle-treated pentylenetetrazole mice (Figure 6).

#### 3.4.2. Biochemical Measurements

*(1) Effects of AFAD on Brain GABA Levels in Pentylenetetrazole-Kindled Mice*. Cortical GABA concentrations decreased significantly in pentylenetetrazole-induced seizure animals, with the highest decrease noted in the vehicle-treated pentylenetetrazole mice. In the negative control mice constituted by vehicle-treated pentylenetetrazole mice, there is a remarkable decrease in the brain GABA concentration from 272.91 ± 75.24 *µ*g/g in the vehicle-treated saline animal to 201.48 ± 38.56 *µ*g/g in the mice challenged by pentylenetetrazole. Oral administration of AFAD at the doses of 80 and 160 mg/kg significantly increased the cortical GABA concentration to 421.18 ± 62.43 *µ*g/g (*P* < 0.05) and 437.24 ± 35.24 *µ*g/g (*P* < 0.01), respectively, compared with vehicle-treated pentylenetetrazole mice. Administration of AFAD produced a significant increase in the brain GABA concentration in mice treated with AFAD at the doses of 80 mg/kg (421.18 ± 62.43 *µ*g/g tissue; *P* < 0.05) and 160 mg/kg (437.24 ± 35.24 *µ*g/g tissue; *P* < 0.01) (Table 2). Interestingly, it was observed that AFAD improved the brain GABA content compared with vehicle-treated saline mice. Sodium valproate administered at a dose of 300 mg/kg exhibited elevated levels of cortical GABA (*P* < 0.01) compared with the vehicle-treated pentylenetetrazole group, but AFAD (20 and 45 mg/kg) do not have a remarkable effect in augmenting GABA levels.

*(2) Effects of AFAD on GABA Transaminase Activity in Pentylenetetrazole-Kindled Mice*. There was a remarkable increase in the levels of GABA transaminase activity in the pentylenetetrazole-kindled mice compared with the normal group (Table 3). Contrarily, the activity of GABA transaminase inhibitory was significantly improved after the AFAD administration at 80 and 160 mg/kg (*P* < 0.05 and *P* < 0.05, respectively) compared with vehicle-treated pentylenetetrazole mice (Table 2). AFAD treatment significantly decreased the brain GABA transaminase activity from 4.52 ± 0.13 units/mg tissue in the vehicle-treated pentylenetetrazole mice to 1.57 ± 0.13 units/mg tissue (*P* < 0.05) and 1.48 ± 0.10 units/mg tissue, respectively, in the groups administered AFAD 80 and 160 mg/kg, respectively. Valproate sodium also significantly restored the levels of reduced glutathione (*P* < 0.05).

*(3) Effects of AFAD on Brain MDA Levels in Pentylenetetrazole-Kindled Mice*. The lipid peroxidation level (malondialdehyde) in the cortex of mice was markedly (*P* < 0.001) increased after the alternative days of pentylenetetrazole treatment; however, pretreatment with 80 and 160 mg/kg AFAD significantly (*P* < 0.001) and dose dependently attenuated pilocarpine-induced increase in thiobarbituric acid-reactive substances in the cortex compared with the vehicle-treated pentylenetetrazole group (Table 3). The MDA levels significantly decreased from 468.31 ± 16.46 nmol/g tissue in the vehicle-treated pentylenetetrazole mice to 192.63 ± 19.51 nmol/g tissue (*P* < 0.05), 169.83 ± 18.17 nmol/g tissue (*P* < 0.01), and 161.49 ± 17.53 (*P* < 0.001) nmol/g tissue in groups administered AFAD 40, 80, and 160 mg/kg, respectively (Table 3).

*(4) Effects of AFAD on Brain Glutathione Levels in Pentylenetetrazole-Kindled Mice*. As can be observed in Table 3, a highly significant (*P* < 0.001) depletion of cortical GSH was observed in vehicle-treated pentylenetetrazole mice and pretreatment with 80 and 160 mg/kg AFAD significantly attenuated pentylenetetrazole-induced depletion in GSH compared with the vehicle-treated pentylenetetrazole group. The brain GSH was significantly lower in the group administered PTZ (114.21 ± 13.34 *µ*g/g tissue; *P* < 0.001) compared with the groups administered vehicle (195.64 ± 15.32 *µ*g/g tissue). Oral administration of AFAD significantly increased the brain GSH levels from 114.21 ± 13.34 *µ*g/g tissue in the vehicle-treated pentylenetetrazole mice to 152.34 ± 16.41 (*P* < 0.05), 176.84 ± 17.65 (*P* < 0.05), and 173.19 ± 14.39 (*P* < 0.01) µg/g tissue in the groups administered AFAD 40, 80, and 160 mg/kg, respectively (Table 3).

*(5). Effects of AFAD on Superoxide Dismutase Activity in Pentylenetetrazole-Kindled Mice*. Treatment of mice with pentylenetetrazole results in decreased levels of brain SOD when compared with vehicle-treated mice. However, our results indicated a significant increase of the SOD-specific activity in pentylenetetrazole-treated groups treated with 40, 80, and 160 mg/kg AFAD compared with the vehicle-treated pentylenetetrazole mice. AFAD treatment significantly increased the brain SOD levels from 58.64 ± 17.74 U/g tissue in the negative control group to 95.79 ± 15.32 (*P* < 0.05) and 116.88 ± 15.34 (*P* < 0.01) U/g tissue in the groups administered 80 and 160 mg/kg AFAD, respectively (Table 3).

*(6). Effects of AFAD on Nitric Oxide Status Content in Pentylenetetrazole-Kindled Mice*. As shown in Table 3, treatment of mice with pentylenetetrazole significantly increased the cortical NO level compared with AFAD administered at the doses of 80 and 160 mg/kg, respectively. AFAD significantly decreased the brain NO levels from 4.19 ± 0.15 µM/mg tissue in the vehicle-treated pentylenetetrazole mice to 1.91 ± 0.23 (*P* < 0.05), 1.64 ± 0.21 (*P* < 0.05), and 1.44 ± 0.17 (*P* < 0.001) *µ*M/mg tissue in the groups administered 40, 80, and 160 mg/kg AFAD, respectively (Table 3).

#### 3.4.3. Effects of AFAD on Pentylenetetrazole-Kindling-Induced Neuronal Cell Loss

**Figure 7:**
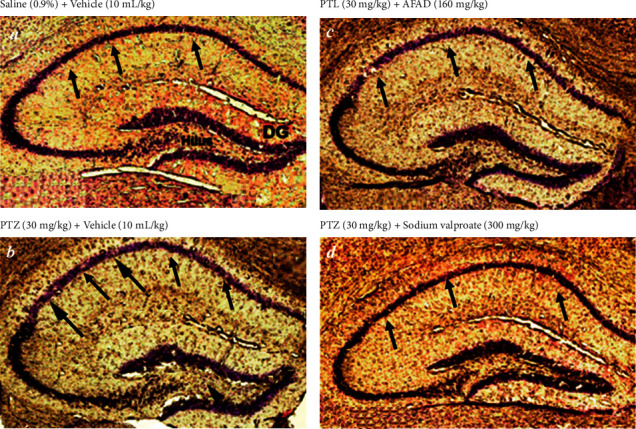
Effects of AFAD or sodium valproate on the pentylenetetrazole-kindling-induced neuronal loss in the hippocampus. Representative images of Nissl staining of hippocampal neurons in vehicle control mice (a), PTZ-challenged mice (b), pentylenetetrazole-challenged 160 mg/kg + AFAD-treated mice (c), and pentylenetetrazole-challenged mice + sodium valproate-treated mice (d). There is neuron loss in the CA1 region and DG hilus of pentylenetetrazole-challenged mice. PTZ, pentylenetetrazole. This figure depicted that extensive neuron loss was detected in the CA1 and the hilar region of the DG (arrows) in PTZ-kindled mice compared with vehicle control mice. Sodium valproate or AFAD significantly ameliorated the neuronal loss (arrows) in the brain of mice. The numbers of stained cells in each field were counted at a higher magnification of ×400.

Here, we examined whether the administration of AFAD can inhibit fully kindled convulsions-induced hippocampal neuron loss in pentylenetetrazole-challenged animals (Figure 7). Neuronal loss was quantified by Nissl staining in hippocampal CA1, CA3, and dentate gyrus subregions. There was a remarkable decrease in the number of neurons in CA1 and DG hilus in the pentylenetetrazole-kindled mice compared with the normal group of mice. However, pentylenetetrazole kindling has not induced a significant reduction in the number of neurons in the CA3 subregion of the brain.

**Figure 8:**
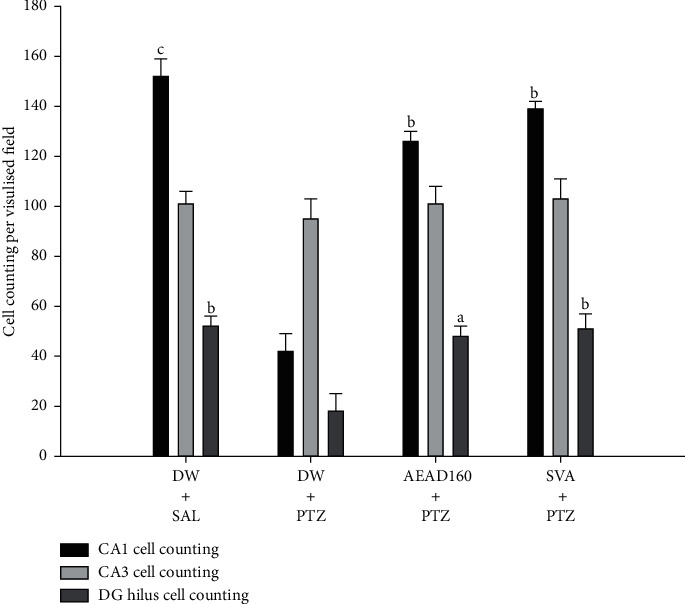
Effects of AFAD or sodium valproate on cell counting per visual field in pentylenetetrazole-kindled mice. Each graph expressed as mean ± S.E.M., presenting quantifications of Nissl staining of neurons in hippocampal CA1, CA3, and DG hilus. Data were analysed by one-way ANOVA followed by Bonferroni's multiple comparison test, ^a^*P* < 0.05, ^b^*P* < 0.01, and ^c^*P* < 0.001, significantly different compared with the pentylenetetrazole-challenged mice. AFAD20, standardised active fractions from *Anthocleista djalonensis* 20 mg/kg; DW, distilled water; PTZ, 30 mg/kg pentylenetetrazole; SVA, 300 mg/kg sodium valproate; SAL, saline. Scale bar = 250 µm. The numbers of stained cells in each field were counted at a higher magnification (×400). The data are presented as the number of cells per high-power field.

Interestingly, administration of 160 mg/kg AFAD significantly ameliorated the neuronal loss in the brain of mice in hippocampal CA1 (*P* < 0.001) and dentate gyrus (*P* < 0.05) subregions (Figure 8). In addition, the effects of AFAD at the doses of 20, 40, and 80 mg/kg were not statistically significant against fully kindled convulsions-induced hippocampal neuron loss and showed very little effects on hippocampal CA1 and dentate gyrus subregions (results are not presented).

### 3.5. Acute Toxicity Study of AFAD

No death was recorded in any of mouse groups treated with the therapeutic doses of AFAD during 14 days of the study. Behavioural parameters of mice were observed following the oral administration of AFAD or distilled water. Signs and symptoms that occurred after the oral administration of 1280 mg/kg AFAD were a reduction of locomotion, and the mice that died from the high doses (1280–7680 mg/kg) of AFAD showed signs of motor and respiratory failure before death. The acute administration of AFAD by oral route at doses up to 7680 mg/kg did not produce death in 50% of animals during 24 hours or 14 days of study. The dose of extract necessary to reduce the response of mice by 50% relative to the negative control value is possible to be more than 7680 mg/kg (Table 4).

## 4. Discussion

These series of experimental models of chemically induced generalised tonic-clonic seizures are looking at interaction of isolated fractions from the root barks of *Anthocleista djalonensis* and some pharmacological targets involved in epileptogenesis or/and epileptic seizures and thereby find out whether these major constituents are active ingredients accounting for activities of *Anthocleista djalonensis*. Animal models of generalised seizures have most frequently been used as tools for screening potential anticonvulsant drugs.

Animals with pentylenetetrazole-induced convulsions represent a common epileptic model for testing antiepileptic drugs with potential for the treatment of epilepsy [46]. In our study, active fractions are selected on the base of bio-guided fractionation and 90 mg/kg pentylenetetrazole-induced generalised tonic-clonic convulsions. Concomitantly, oral administration of AFAD or the standard antiepileptic drug (clonazepam) induced a significant protection of mice in pentylenetetrazole tests. The strong antagonism of convulsions induced by 90 mg/kg pentylenetetrazole suggested the existence of anticonvulsant activities of AFAD and its interaction with the GABA complex receptors [46, 47]. The obtained results suggested that the effects of AFAD are mediated by central synapses employing GABA as an inhibitory transmitter [48]. Our results indicated the presence of some compounds from the classes of iridoid glucoside and xanthone, which can support the biological activity of AFAD. These effects may correlate with the presence of iridoid glucoside (sweroside) [8, 49] and ursolic acid (xanthone) [50, 51] in the AFAD. Elsewhere, similar results were obtained in previous studies where an extract containing a significant amount of ursolic acid and compounds from the class of iridoid glucoside demonstrated strong anticonvulsant and sedative properties by activation of the GABAergic neurotransmitter system [50, 51].

Previous investigation indicated that seizures rely on the evidence of a paroxysmal, stereotypical behaviour that disrupts normal function and has characteristic electroclinical correlate by electroencephalographic or electrophysiological studies. Real-time video-electroencephalographic recording in the animals that are alive has become the gold standard for evaluating seizure generation and its underlying mechanisms in laboratory studies of epilepsy [52, 53]. Intraperitoneal injection of pentylenetetrazole not only produces epileptiform activity in animals but also mimics seizures-induced behavioural changes that are very identical to grand mal epilepsy with the main characteristics observed in humans. The present study demonstrated a potent anticonvulsant activity of AFAD against seizures-induced pentylenetetrazole. Specifically, AFAD strongly inhibited the behavioural disorders and electroencephalographic alterations due to pentylenetetrazole injection in mice. Antagonism of hippocampal and cortical paroxysmal discharges by oral administration of AFAD is characterised by the significant reduction of the number of crisis, the cumulative duration of crisis, and the mean duration of crisis [54]. These results indicate that the inhibition of specific excitatory drive in the cortex and hippocampus is the consequence of the delay of latency to seizures and concomitantly the inhibition of epileptiform discharges in the hippocampus and cortex. This provides a proof of the principle that AFAD could be used for the treatment of generalised tonic-clonic seizures and for management of generalised epilepsy [8, 46, 55].

Pretreatment of mice with AFAD indicated a significant and dose-dependent protection against kindled seizures induced by pentylenetetrazole. Oral administration of AFAD increased the latencies to myoclonic jerks and clonic seizures as well as the latencies to generalised seizure. The active fractions significantly reduced the number of seizures and ameliorated behavioural seizures in pentylenetetrazole-challenged mice. These results are similar to those reported in our previous research in which *Anthocleista djalonensis* extracts protected mice against seizures induced by pentylenetetrazole [27]. Pentylenetetrazole-induced kindling is an experimental model of epilepsy in which chronic chemostimulation of the central nervous system pathway induces a spontaneous hyperexcitable state (kindling and fully kindled seizures, as well as spike waves epilepsy) and well-established generalised tonic-clonic seizures, providing a good evidence to study epileptic disorders [10, 56]. The inhibition of pentylenetetrazole-induced seizures suggested the existence of anticonvulsant properties that might involve an action on benzodiazepine and/or GABA sites [57]. Several studies reported that iridoid glucoside and ursolic acid were concerned with the drug interference caused by some antiepileptic medicinal plants [8, 50]. The presence of these compounds in AFAD suggested their merit on inducing the anticonvulsant properties in pentylenetetrazole-kindled mice.

Several mechanisms underlying such effects of *Anthocleista djalonensis* were previously proposed [27]. There were two such mechanisms: (1) *Anthocleista djalonensis* enhances gamma-aminobutyric acid (GABA)-mediated inhibition, reduces repetitive firing, and reduces both inhibition and excitation in neuronal networks and (2) *Anthocleista djalonensis* enhances the inhibitory action of GABA, decreases inhibition in neuronal networks, and affects calcium ion transport with low activities on repetitive firing [27, 58]. The results indicated that AFAD significantly increased the GABA level in the brain tissue identically to those of sodium valproate administered at a dose of 300 mg/kg. Enhanced GABA-mediated inhibition is a major mechanism for preventing or arresting clinical and experimental seizures, and there is strong electrophysiological, pharmacological, and biochemical evidence that many of the antiepileptic drugs in common clinical use (e.g., benzodiazepine, barbiturates, and valproate) partially exert their anticonvulsant action through interaction with the GABAergic signalling system. Its reduction in the brain is associated with a number of neurological disorders such as epilepsy and anxiety [59, 60]. These results suggested that the anticonvulsant action of AFAD is correlated to an increase in the GABA level in the brain [56, 61]. Interestingly, other results performed on the central nervous system-depressant activity of monoterpenes indicated the increase of the total sleep time in the pentobarbital-induced hypnosis model in mice, indicating a sedative property [62]. Compounds from the class of monoterpene diol identified in AFAD may justify the presence of anticonvulsant properties in this standardised fraction. These properties may be attributed to an action on the central mechanisms involved in the regulation of the GABAergic neurotransmission system.

Gamma-aminobutyrate aminotransferase known as GABA-T is a catabolic enzyme that catalyses the transfer of the amino group from gamma-aminobutyrate to 𝛼-ketoglutarate, leading to the reduction of the GABA level [63, 64]. Inhibition by sodium valproate of the GABA-metabolising enzymes, GABA-transaminase and succinic semialdehyde dehydrogenase, can be demonstrated in rodents [65]. Our results indicated that the injections of pentylenetetrazole significantly increased the level of GABA-T activity in the mice brain. However, oral administration of AFAD strongly attenuated the enzyme activity, indicating potent GABA-T inhibitory activity. These results confirm the increase of brain GABA content in pretreated mice with the *Anthocleista djalonensis* standardised fraction [66].

The seizure activity during epileptogenesis and/or epilepsy decreases the antioxidant defence mechanism in the brain and increase the amount of free radicals, which further induces the oxidative stress [67]. In the present study, there is a significant increase of brain lipid peroxidation in the mice challenged with pentylenetetrazole, which indicated the excess production of free radicals and oxidative stress in the brain [68]. Administration of AFAD significantly prevented the increase in brain malondialdehyde levels (an indicator of the lipid peroxidation due to free radicals); probably, this reduction was evident in the whole brain. Therefore, it may be concluded that antiepileptic effects of AFAD is partly mediated through its antioxidant property. The results of protection offered by AFAD were comparable with those of the sodium valproate-treated group. Thus, it can be hypothesised that a reduction in the product of lipid peroxidation could partly contribute to the protective effects of AFAD and sodium valproate in the studied model.

The glutathione redox system is a very efficient free radical scavenger and involved in the protection of the central nervous system from the toxic effects of reactive oxygen compounds (hydroxyl, alcoxyl, peroxyl, superoxide, nitric oxide, and nitrogen dioxide) basically derived from oxygen [69]. Recent studies suggest that reduced glutathione may be causally involved in various neurological disorders such as epilepsy and ischaemia-reperfusion injury; and the differences are reported in free radical scavenging enzyme levels [70]. Intraperitoneal injection of pentylenetetrazole significantly decreased the level of glutathione in kindled mice brain tissue. This oxidised form of glutathione reacts with free radicals and prevents the generation of most toxic hydroxyl radicals [71]. Interestingly, in our study, AFAD has restored the level of reduced glutathione in pentylenetetrazole-challenged mice.

Superoxide dismutase is an enzyme catalysing the reaction of superoxide radical disproportionation and thereby reducing oxidative stress in the brain. The reduction of this enzyme activity could result in the inhibition of removal of superoxide ions and hydrogen peroxide radicals, which brings about a number of reactions that are harmful to the neuronal tissue [72]. The decrease in the activities of this enzyme could be due to increased reactive oxygen free radicals, which can themselves reduce the activity of this enzyme. This is in agreement with our findings where pentylenetetrazole-administered mice showed decreased activities of superoxide dismutase. The mice administered AFAD showed increased activity of this enzyme, which suggests that AFAD may have ability to reduce or prevent the toxic effects induced by free radicals. Previous studies demonstrated that intra-amygdala injection of exogenous superoxide dismutase has been shown capable of reducing the brain's convulsive activity resulting from kindling [68]. This forms the basis for the neuroprotective effects of AFAD administration.

Injections of pentylenetetrazole induced a significant increase in nitric oxide levels in kindled animals after chronic treatment. Induction of kindling induced the increase of neuronal nitric oxide synthase activity, and its pathway is related to activation of the NMDA receptor [73]. However, the reference drug sodium valproate or AFAD significantly decreased the nitric oxide level. Pentylenetetrazole via (N-methyl-D-aspartate) NMDA glutamate receptors activates calcium release via NMDA receptors that consequently activates the calcium-calmodulin pathway to increase neuronal nitric oxide protein expression, and the nitric oxide concentration is able to induce spontaneous occurrence of grand mal epilepsy and/or temporal lobe epilepsy. Reduction of nitric oxide levels induced by AFAD may indicate that nitric oxide suppression activity is partly responsible for its antiepileptogenic effects [74]. A plethora of studies, however, suggest that ursolic acid, iridoid glucoside, and pentacyclic triterpene have antioxidant activities through upregulation of antioxidant defence in experimental animals. Eventually, through the combined anticonvulsant, antioxidant, and neuroprotective mechanisms, these isolated compounds from AFAD are endowed with a unique potential to ameliorate a range of neuronal cell loss [8, 75].

It is established that the chronic injection of pentylenetetrazole produced a gradual increase of severe seizures accompanied by oxidative stress and lead to cell loss in various regions of the brain especially in the hippocampal CA1, CA3, and dentate gyrus subregions [42, 76]. As can be indicated in our results, there was a significant decrease in the number of neurons in CA1 and DG hilus in the pentylenetetrazole-kindled mice. Neuronal damages were quantified by Nissl staining in the different regions of the brain [77]. Interestingly, the oral administration of AFAD in mice significantly antagonised the deleterious effects of pentylenetetrazole kindling on a number of neurons in the CA1 and DG hilus. On the other hand, our results indicated the increase of the product of lipid peroxidation in the brain with a dramatic reduction in antioxidant enzymatic activities accompanied with an obvious neuron loss in the hippocampal CA1 and DG hilus regions, indicating that pentylenetetrazole kindling significantly induced oxidative stress. Accompanied with this oxidative damage, we observed an obvious neuron loss in the hippocampal CA1 and DG hilus regions. We suspect that this hippocampal neuron loss might be caused by pentylenetetrazole-kindling-induced oxidative stress [42]. Treatment with a higher dose of AFAD strongly suppressed pentylenetetrazole-kindling-induced oxidative stress as well as neuron loss in the hippocampal CA1 and DG hilus regions. This suggests that the administration of AFAD ameliorates oxidative stress and neuronal cell death processes in this pentylenetetrazole-kindling epilepsy model and finally leads to neuroprotection.

No adverse effects and mortality were observed in the acute toxicity test of AFAD (range studied, 20–640 mg/kg). Therefore, the present result confirms, in part, the safety of AFAD at the therapeutic doses in mice.

## 5. Conclusions

In conclusion, AFAD has shown a protective effect against pentylenetetrazole-induced generalised tonic-clonic convulsions possibly through the enhancement of cortical antioxidant defence, GABAergic signalling, and the reduction of neuronal cell loss.

More studies are required to develop it as an add-on therapy for the comprehensive management of grand mal epilepsy. The pharmacological and chemical studies are continuing in order to characterise the mechanism(s) responsible for this anticonvulsant and antioxidant action and also to identify the active substances present in the root barks of *Anthocleista djalonensis*.

As a limitation for this work, the effects of AFAD on mice models of mesiotemporal lobe epilepsy without generalisation of seizures in the brain but with nonconvulsive focal seizures were not evaluated since the primary aim of our study is to identify only the effects of AFAD on pentylenetetrazole models of generalised tonic-clonic convulsions, biochemical changes, and neuronal cell loss.

## Figures and Tables

**Table 1:** Effects of *Anthocleista djalonensis* pooled fractions, standardised active fractions from *Anthocleista djalonensis* (AFAD), or clonazepam on acute administration of pentylenetetrazole-induced convulsions.

Groups	Dose (mg/kg)	Latency to convulsion (min)	% Exhibiting convulsions	Latency to death (min)	% Surviving animals
Vehicle + PTZ	– + 90	03.27 ± 01.13	100	06.14 ± 1.17	00
FPool 1 + PTZ	80 + 90	03.91 ± 01.17	100	05.61 ± 1.41	00
FPool 2 + PTZ	80 + 90	04.83 ± 01.14	100	06.74 ± 1.91	00
FPool 3 + PTZ	80 + 90	05.18 ± 01.24	100	07.12 ± 1.25	00
FPool 4 + PTZ	80 + 90	04.43 ± 02.17	100	07.76 ± 2.07	00
FPool 5 + PTZ	80 + 90	20.84 ± 01.82^*∗∗*^	20^*∗∗*^	21.17 ± 2.18^*∗∗∗*^	70^*∗∗*^
FPool 6 + PTZ	80 + 90	04.28 ± 01.34	100	08.25 ± 2.67	00
FPool 7 + PTZ	80 + 90	07.15 ± 01.57^*∗*^	70	11.94 ± 2.92^*∗*^	20
FPool 8 + PTZ	80 + 90	05.41 ± 01.34	00	08.05 ± 1.75	00
FPool 9 + PTZ	80 + 90	04.49 ± 02.21	00	07.31 ± 1.19	00
FPool 10 + PTZ	80 + 90	03.92 ± 01.69	100	05.34 ± 1.24	00
AFAD + PTZ	20 + 90	05.57 ± 01.34	80	12.81 ± 2.09^*∗*^	30
AFAD + PTZ	40 + 90	12.91 ± 03.27^*∗∗*^	40^*∗*^	25.61 ± 3.48^*∗*^	60^*∗*^
AFAD + PTZ	80 + 90	21.64 ± 03.81^*∗∗∗*^	20^*∗∗*^	23.09 ± 2.91^*∗∗*^	70^*∗∗*^
AFAD + PTZ	160 + 90	24.73 ± 02.14^*∗∗∗*^	10^*∗∗∗*^	29.15 ± 2.67^*∗∗∗*^	90^*∗∗∗*^
Clonaz + PTZ	0.1 + 90	26.71 ± 01.19^*∗∗∗*^	10^*∗∗∗*^	27.62 ± 1.26^*∗∗∗*^	90^*∗∗∗*^

Results are expressed as mean ± S.E.M. for 10 animals. Data were analysed by one-way ANOVA followed by Bonferroni's multiple comparison test, ^*∗*^*P* < 0.05,  ^*∗∗*^*P* < 0.01,  ^*∗∗∗*^*P* < 0.001, significantly different compared with the pentylenetetrazole-challenged mice. FPool 5 or AFAD, standardised active fractions from *Anthocleista djalonensis*; Clonaz, 0.1 mg/kg clonazepam; PTZ, 90 mg/kg pentylenetetrazole.

**Table 2:** Effects of AFAD or sodium valproate on brain GABA content and GABA transaminase activity in the whole mice brain after chronic administration of pentylenetetrazole-induced kindling.

Group	Dose (mg/kg)	GABA level in brain tissue (µg/g tissue)	GABA-T activity in brain tissue (units/mg tissue)
Vehicle + saline	– + –	272.91 ± 75.24^*∗*^	2.02 ± 0.11^*∗*^
Vehicle + PTZ	– + 90	201.48 ± 38.56	4.52 ± 0.13
AFAD + PTZ	20 + 90	217.42 ± 91.18	3.41 ± 0.12
AFAD + PTZ	40 + 90	221.68 ± 17.54	3.13 ± 0.12
AFAD + PTZ	80 + 90	421.18 ± 62.43^*∗*^	1.57 ± 0.13^*∗*^
AFAD + PTZ	160 + 90	437.24 ± 35.24^*∗∗*^	1.48 ± 0.10^*∗*^
SVA + PTZ	300 + 90	301.15 ± 27.47^*∗∗*^	2.26 ± 0.12^*∗*^
AFAD + saline	160 + –	299.68 ± 16.25^*∗∗*^	2.14 ± 0.11^*∗*^

Results are expressed as mean ± S.E.M. for 10 animals. Data were analysed by one-way ANOVA followed by Bonferroni's multiple comparison test, ^*∗*^*P* < 0.05,  ^*∗∗*^*P* < 0.01, significantly different compared with the pentylenetetrazole-challenged mice. AFAD, standardised active fractions from *Anthocleista djalonensis*; SVA, 300 mg/kg sodium valproate; PTZ, 30 mg/kg pentylenetetrazole.

**Table 3:** Effects of AFAD or sodium valproate on markers of oxidative stress and nitric oxide status in the whole mice brain after chronic administration of pentylenetetrazole-induced kindling.

Group	Dose (mg/kg)	MDA (nmol/g tissue)	GSH (µg/g tissue)	SOD (U/g tissue)	NO (µM/mg tissue)
Vehicle + saline	– + –	153.17 ± 18.72^*∗∗∗*^	195.64 ± 15.32^*∗∗∗*^	107.75 ± 14.35^*∗∗*^	2.99 ± 0.12^*∗*^
Vehicle + PTZ	– + 85	468.31 ± 16.46	114.21 ± 13.34	58.64 ± 17.74	4.19 ± 0.15
AFAD + PTZ	20 + 85	378.49 ± 21.98	127.11 ± 17.42	71.15 ± 16.42	2.97 ± 0.21
AFAD + PTZ	40 + 85	192.63 ± 19.51^*∗*^	152.34 ± 16.41^*∗*^	75.56 ± 11.09^*∗*^	1.91 ± 0.23^*∗∗*^
AFAD + PTZ	80 + 85	169.83 ± 18.17^*∗∗*^	176.84 ± 17.65^*∗*^	95.79 ± 15.32^*∗*^	1.64 ± 0.21^*∗∗*^
AFAD + PTZ	160 + 85	161.49 ± 17.53^*∗∗∗*^	173.19 ± 14.39^*∗∗*^	116.88 ± 15.34^*∗∗*^	1.44 ± 0.17^*∗∗∗*^
SVA + PTZ	300 + 85	157.28 ± 16.82^*∗∗∗*^	185.19 ± 15.14^*∗∗*^	121.17 ± 19.35^*∗∗*^	1.57 ± 0.14^*∗∗∗*^
AFAD + saline	160 + –	159.61 ± 19.25^*∗∗∗*^	199.41 ± 17.28^*∗∗∗*^	119.61 ± 12.09^*∗∗*^	2.89 ± 0.13^*∗∗∗*^

Results are expressed as mean ± S.E.M. for 10 animals. Data were analysed by one-way ANOVA followed by Bonferroni's multiple comparison test. ^*∗*^*P* < 0.05,  ^*∗∗*^*P* < 0.01,  ^*∗∗∗*^*P* < 0.001, significantly different compared with the pentylenetetrazole-challenged mice. AFAD, standardised active fractions from *Anthocleista djalonensis*; PTZ, 30 mg/kg pentylenetetrazole; SVA, 300 mg/kg sodium valproate; MDA, lipid peroxidation; GSH, reduced glutathione; SOD, superoxide dismutase; NO, nitric oxide.

**Table 4:** Acute toxicity of AFAD administered orally to different groups of male and female mice.

Group	Dose (mg/kg)	Sex	Dead/Treated mice	Mortality latency (h)	Toxic symptoms
Vehicle	—	Male	0/5	—	None
	—	Female	0/5	—	None

AFAD	20	Male	0/5	—	None
		Female	0/5	—	None

AFAD	40	Male	0/5	—	None
		Female	0/5	—	None

AFAD	80	Male	0/5	—	None
		Female	0/5	—	None

AFAD	160	Male	0/5	—	None
		Female	0/5	—	None

AFAD	320	Male	0/5	—	None
		Female	0/5	—	None

AFAD	640	Male	0/5	—	None
		Female	0/5	—	None

AFAD	1280	Male	1/5	36–48	Hypoactivity, salivation
		Female	1/5	36–48	Hypoactivity, salivation

AFAD	2560	Male	1/5	36–48	Hypoactivity, salivation
		Female	1/5	36–48	Hypoactivity, salivation

AFAD	5120	Male	1/5	36–48	Hypoactivity, piloerection, salivation
		Female	1/5	36–48	Hypoactivity, piloerection, salivation

AFAD	7680	Male	2/5	36–48	Hypoactivity, asthenia, anorexia, salivation
		Female	1/5	36–48	Hypoactivity, asthenia, anorexia, salivation

None = no toxic symptoms during the observation period; mortality latency = time to death after the oral administration. Mice in each group were carefully examined for any signs of toxicity (behavioural changes and mortality) for 14 days. The control group received vehicle (10 mL/kg, *per os*). AFAD, standardised active fractions from *Anthocleista djalonensis*.

## Data Availability

All the data used to support the findings of this study are included within the article, and the results presented are carried out by the authors. The data used as references were properly cited.

## Abbreviation

ANOVA: Analysis of variance
^13^C NMR: Carbon-13 nuclear magnetic resonance
Clonaz: Clonazepam
ED_50_: Median effective dose
EEGs: Electroencephalograms
GABA: Gamma-aminobutyric acid
GABA-T: Gamma-aminobutyric acid transaminase
GSH: Reduced glutathione
HPLC: High-performance liquid chromatography
^1^H-NMR: Proton nuclear magnetic resonance
LD50: Median lethal dose
MDA: Malondialdehyde
PTZ: Pentylenetetrazole
S.E.M.: Standard error of the mean
SOD: Superoxide dismutase
SSA: Succinic semialdehyde
SVA: Sodium valproate.

## Supplementary Materials

Supplementary Materials S1: animal study authentication.
