# Supplementary material for: Protective Effects of Anthocleista djalonensis Extracts against Pentylenetetrazole-Induced Epileptic Seizures and Neuronal Cell Loss: Role of Antioxidant Defense System
Source: Evid Based Complement Alternat Med. 2021 Aug 30;2021:5523705. doi: 10.1155/2021/5523705 (PMC8423543; doi:10.1155/2021/5523705)
Supplement: Supplementary Materials — S1: animal study authentication. [file 5523705.f1.pdf]

UNIVERSITY OF BUEA

P.O. Box 63,
Buea, CAMEROON
Tel: (237) 233 32 21 34/ 233 32 46 90/233 32 47 13
Fax: (237) 233 32 22 79

REPUBLICOF CAMEROON
PEACE – WORK – FATHERLAND

UNIVERSITY OF BUEA-INSTITUTIONAL ANIMAL CARE AND USE COMMITTEE (UB-IACUC)

Credited by Decision No. 2016/0877/UB/BTU/VC of 23rd September 2016

Chair: Associate Professor Jane-Francis Akoachere
Secretary: Dr Rene Bilingwe Ayiseh

Your Ref: ____________________

Our Ref: 2019/ 002 / UB/IACUC/BTU/FS

Date: 30-01-2019

Notice of UB-IACUC Approval

Application number: *017*

Permit number: *UB-IACUC N° 002/2019*

Principal Investigator: *Bila Raymond Bess*

Study title: *Neuroprotective effects of Cymbopogon citratus, Anthocleista djalonensis, and Crassocephalum bauchiense extracts in mice models of epileptic seizures and cerebral malaria.*

Application type: ***Initial***

Sponsor: ***Student***

Date of Approval: ***30/01/2019***

Duration of Approval: ***1year***

Principal Investigator (PI)'s responsibilities:

1. The study must be conducted in strict accordance with the protocol approved by the Committee.
2. Changes to the protocol must be submitted to the Committee using the UB-IACUCMinorAmendmentForm for approval before implementation.
3. The PI is responsible for the conduct of the study. The study must be implemented according to national and international guidelines for the ethical conduct of animal research. The PI must collaborate with the Committee monitoring the implementation of the protocol. The Committee has the right to carry out in promptus visits to your animal facility/site of experiment after approval of your protocol.
4. Any future correspondence must include the application number and the PI's name in the subject line.
5. A renewal application or project closure report must be submitted to the Committee at least one month prior to the expiration. This must be done using the UB-IACUC PI's report back form in hard copy at the secretariat in the Biotechnology Unit and via email to ubiacuc@gmail.com making reference to your application number.

University of Buea Institutional Animal Care and Use Committee UB-IACUC Secretariat

Dr. Rene B. Ayiseh
Secretary, UB-IACUC
Faculty of Science, University of Buea

Assoc. Prof. Jane-Francis Akoachere
Chair, UB-IACUC
Faculty of Science, University of Buea
